# Period2 downregulation inhibits glioma cell apoptosis by activating the MDM2-TP53 pathway

**DOI:** 10.18632/oncotarget.8439

**Published:** 2016-03-28

**Authors:** Niu Zhanfeng, Wang Chengquan, Xia Hechun, Wang Jun, Zhang Lijian, Ma Dede, Liu Wenbin, Yin Lei

**Affiliations:** ^1^ Department of Neurosurgery, The General Hospital of Ningxia Medical University, Yinchuan, 750004, China; ^2^ Incubation Base of National Key Laboratory for Cerebrocranial Diseases, Ningxia Medical University, Yinchuan, 750004, China; ^3^ The People's Hospital of Liaocheng City, Liaocheng, 252000, China; ^4^ Ningxia Medical University, Yinchuan, 750004, China; ^5^ Department of ICU, The General Hospital of Ningxia Medical University, Yinchuan, 750004, China

**Keywords:** period2, glioma, TP53, X-ray, U343 cells

## Abstract

The Period2 (Per2) gene is an essential component of the mammalian circadian clock and is strongly linked to glioma occurrence and its response to radiotherapy. Here, we examined the role of Per2 in the response to X-ray-induced DNA damage in U343 glioma cells and in a mouse cancer model. Following low dose X-ray irradiation, we observed that lowering Per2 expression using RNAi reduces DNA damage and cell death in U343 cells and glioma tissue. Additionally, Per2 was associated with increased TP53 activity and was involved in the DNA damage during TP53-mediated apoptosis. These findings suggest that Per2, a core circadian gene, is not only a tumor suppressor gene but can also be regarded as an upstream regulator of TP53. It thus appears that Per2 is an important inhibitor of tumor growth that acts by increasing TP53 expression, DNA damage repair, and apoptosis.

## INTRODUCTION

Gliomas are amongst the most lethal forms of cancer [1, 2]. Their invasive growth makes complete tumor resection very difficult leading to high lethality [3]. A better molecular understanding of adjuvant therapies for glioma could lead to new, more effective therapeutic approaches to treat gliomas.

The Period2 (Per2) gene, an indispensable component of the mammalian circadian clock [4], has been reported to play an important role in tumorigenesis. Per2-deficient mice show increased tumor development [5] and Per2 is often elevated in tumor but not in peripheral tissues [6–10]. Overexpression and/or mutations in the Per2 gene correlate with enhanced tumor growth in breast cancer, colon cancer, and lymphoma, along with altered expression of TP53 and the oncogenes BCLxl, BCL-2, cyclinB1, cyclin D, cyclin E, and c-myc [11–16]. Per2 has been linked to DNA damage response pathways [17], and low Per2 expression may increase the efficacy of radiotherapy against glioma by promoting apoptosis [18–20].

The transcription factor p53 is known as the “guardian of the genome” [21, 22]. The majority of human tumors have mutations in the TP53 gene, which encodes the p53 protein [23]. Additionally, the p53 tumor suppressor is a key transcription factor that regulates cellular pathways, such as DNA repair, cell cycle, apoptosis, angiogenesis, and senescence. p53 acts as an important defense mechanism against cancer onset and progression and is negatively regulated by interactions with the oncoprotein murine double minute 2 (MDM2). In human cancers, the TP53 gene is frequently mutated or deleted or wild-type p53 function is inhibited by high levels of MDM2, leading to downregulation of the tumor suppressive p53 pathways. Thus, the inhibition of MDM2-p53 interactions presents an appealing therapeutic strategy for the treatment of glioma. Recent studies show that the MDM2-p53 interaction is complex, involving multiple levels of regulation by numerous cellular proteins and epigenetic mechanisms. A comprehensive re-examination of this intricate interplay is imperative [24].

We previously reported that Per1 and Per2 expression abnormalities are associated with glioma occurrence [9]. Additionally, decreased Per1 and Per2 expression increases the efficacy of radiotherapy against glioma by promoting apoptosis [20, 25]. Here, we examine the role of Per2 in X-ray-induced DNA damage in U343 glioma cells and an animal glioma model.

## RESULTS

### Expression of per2 in U343 glioma cells

**Figure 1:**
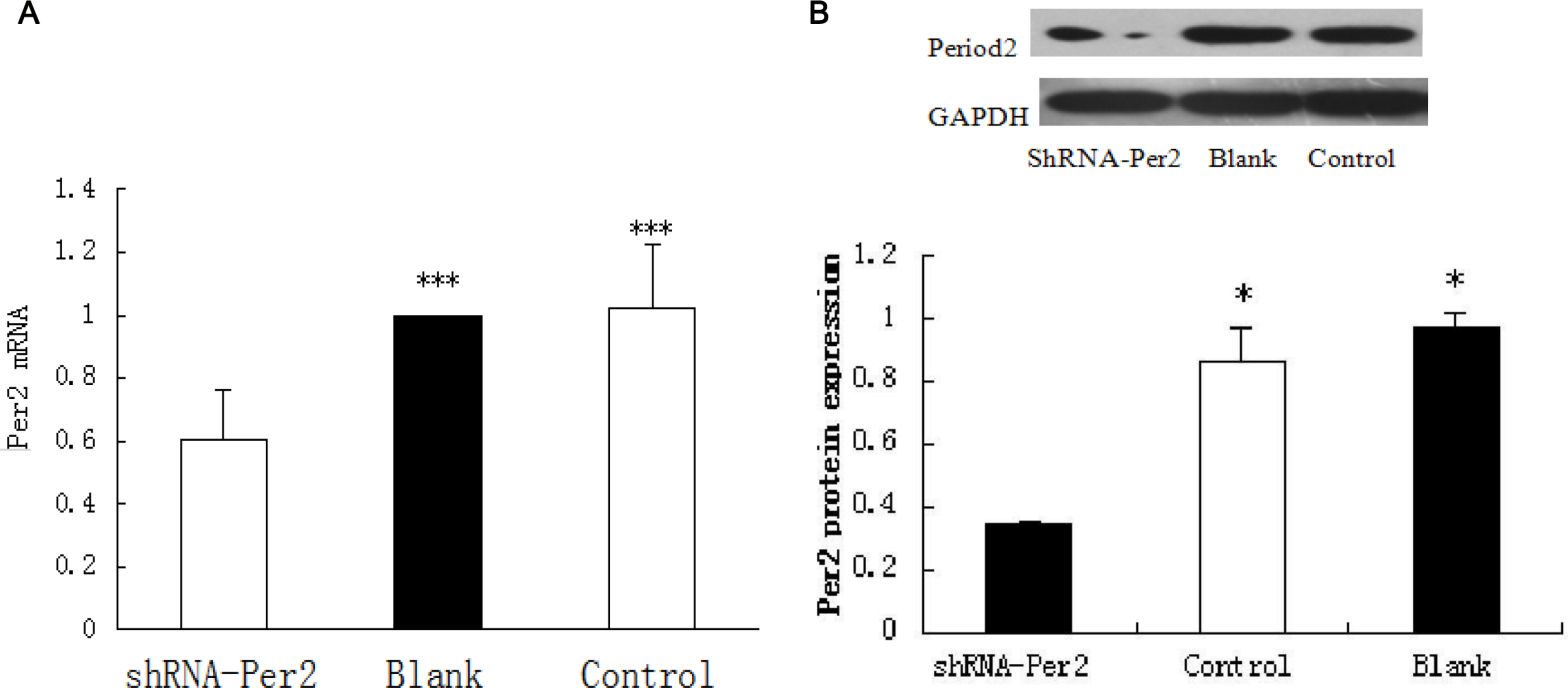
Per2 expression in sh-Per2 treated and two control groups of U343 glioma cells **(A)** mRNA was measured by qRT-PCR with Period2 primers and **(B)** protein was measured by Western blot with antibodies against period2. Cleaved GAPDH was used as an internal control. Significance was determined with a one-way ANOVA with Bonferroni post-test: ***p < 0.001,*p < 0.05.

We determined Per2 mRNA and protein expression levels in the U343MG cell line, which overexpresses MDM2 and maintains wild-type p53 levels, using quantitative real-time PCR (qRT-PCR) and Western blotting, respectively. Both Per2 mRNA and protein levels were notably reduced in the U343 cells transfected with shRNA-PER2 relative to the shRNA-transfected control U343 cells (*P* < 0.05; *n* = 3) or blank-treated U343 cells(the blank treatment is the normal U343 cells) (*P* < 0.05; *n* = 3; Figure 1).

### Correlation between Per2 expression and glioma growth

**Figure 2:**
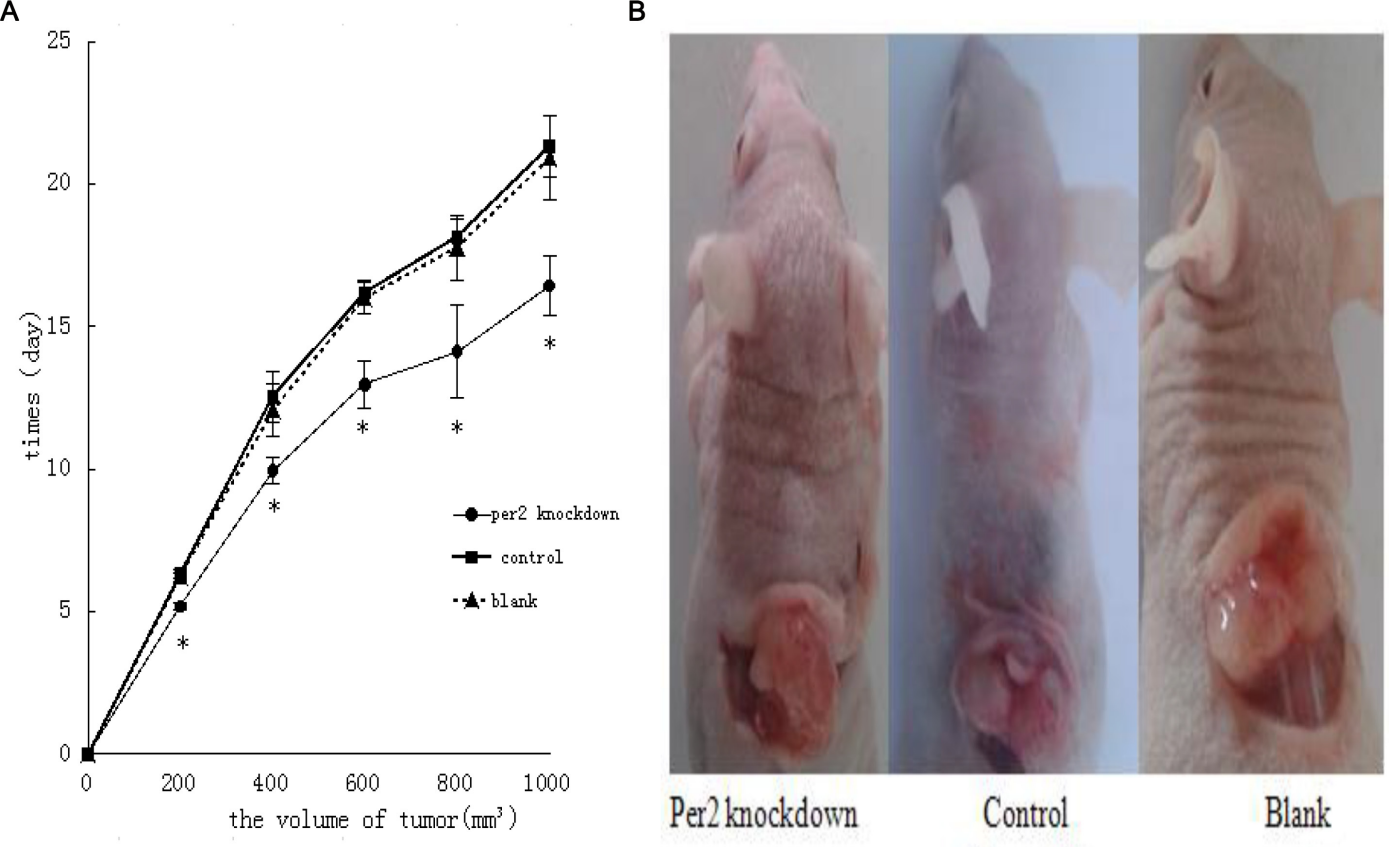
Effect of Per2 on U343 tumor growth in nude mice **(A)** Per2-deficient U343 human glioma xenografts were established in male athymic nude mice; negative controls were treated with contol-virus or blank U343 human glioma cells. Tumor volume was measured daily after treatment. Results are expressed as means ± SEM (each group, *n* = 18). *p < 0.05 **(B)** Each group reached the standard volume. Volume calculation method: We measured the length (a), width (b), and height (l) of each tumor and used the formula: V(volume) = V = abl × π/6. When the volume of each group increased by 200 mm3, we recorded the time. We irradiated each tumor with 10 Gy X-ray until the size reached the standard volume (1000 mm3).

**Figure 3:**
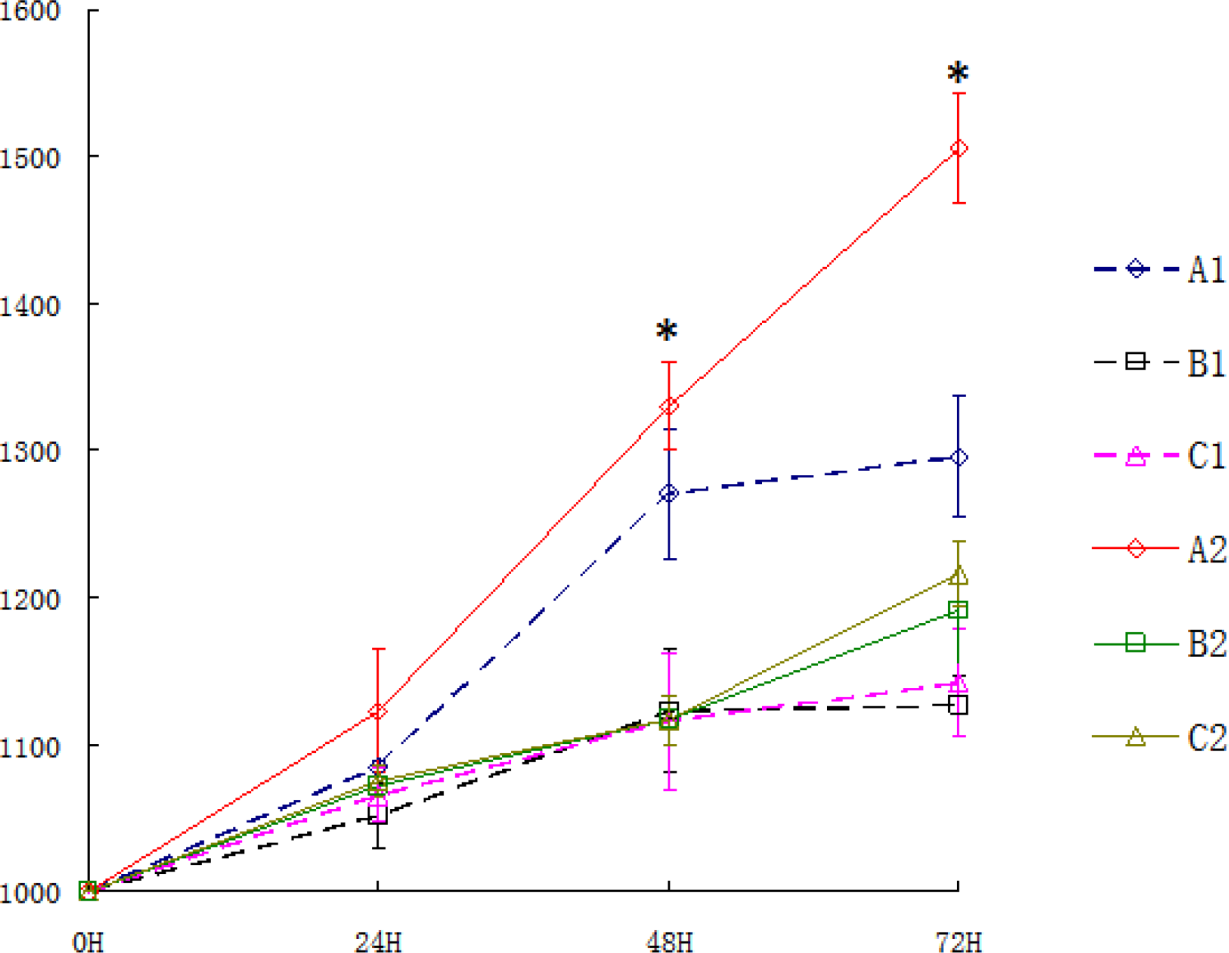
The volume of tumors after X-ray irradiation in each group A1 : Per2 knockdown group with ionizing radiation; B1: Blank group with ionizing radiation; C1: Control group with ionizing radiation; A2: untreated Per2 knockdown group; B2: untreated Blank group; C2: untreated control group.

We injected three types of U343MG cells (2 × 107 cells) into the dorsolateral region of nude mice, and tumors grew in approximately 95% of the mice within 2–3 weeks. We found that tumor growth in the Per2-deficient group was substantially faster than the control virus-treated group or the blank-treated group (both, *P* < 0.05). Additionally, we observed that the tumors in the Per2-deficient group reached a standard volume (1000 mm3) earlier than those in the other two groups (Figure 2A and 2B). When the volume of each group reached the standard volume (1000 mm3), they were exposed to 10 Gy X-ray. We recorded the volume of each group at 24, 48, and 72 hours after irradiation. After 24 hours the 3 groups were indistinguishable but by the 48 and 72 hour time points, the Per-2 deficient mice had larger tumors than either of the two control groups (Figure 3).

### Effect of irradiation on Per2 gene expression

**Figure 4:**
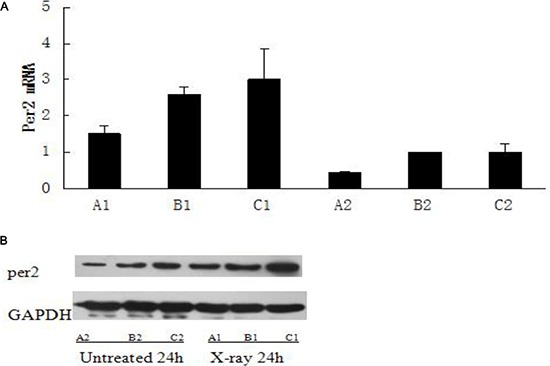
The expression of Per2 in untreated and X-ray treated groups on ZT24 (**A1**): Per2 knockdown group with ionizing radiation at ZT24; (**B1**): Blank group with ionizing radiation at ZT24; (**C1**): Control group with ionizing radiation at ZT24; (**A2**): untreated Per2 knockdown group at ZT24; (**B2**): untreated Blank group at ZT24; (**C2**): untreated control group at ZT24. Results were analyzed by Western blot with antibodies against period2 and qRT-PCR with primer of Period2, and cleaved GAPDH was used as an internal control. Significance was determined with a one-way ANOVA with Bonferroni post-test: *p < 0.05.

**Figure 5:**
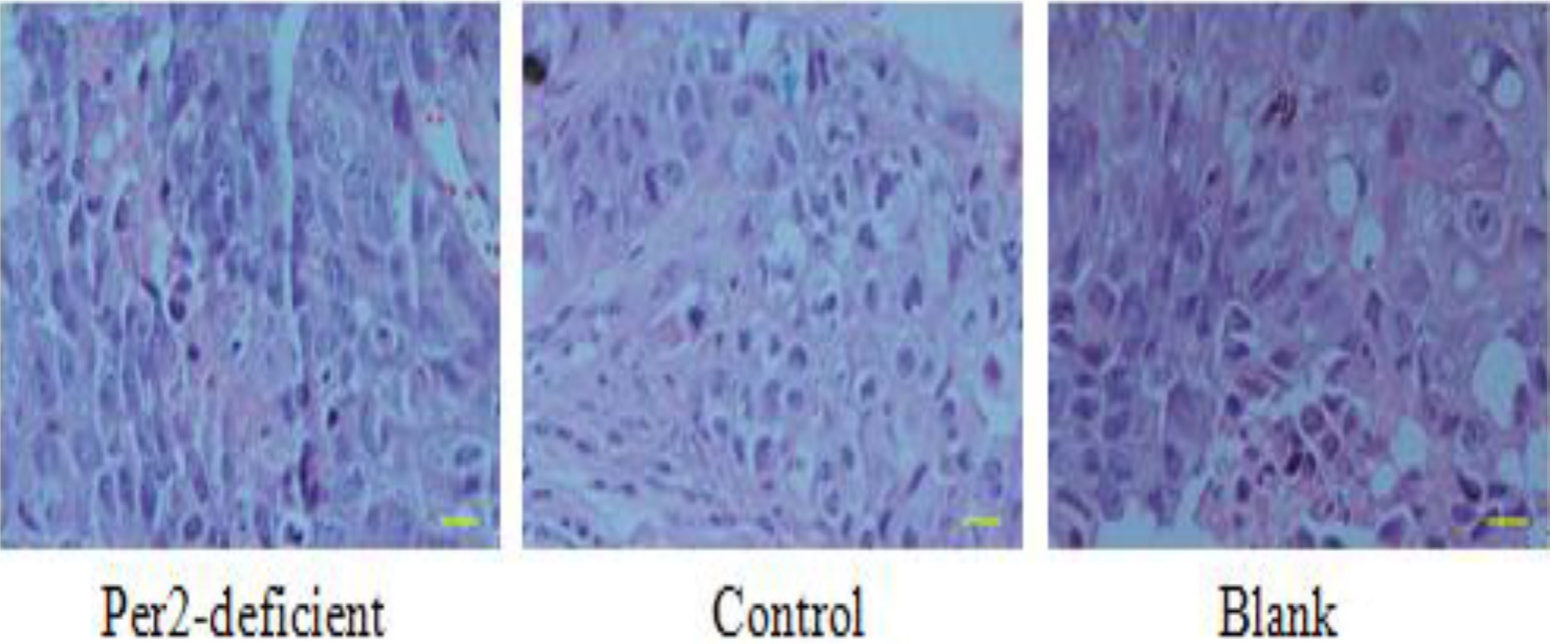
From H & E staining, cells exhibited nuclear atypia and tumor-like morphology H & E staining showed blue and pink speckles representing cell nucleus and cytoplasm, respectively. Scale bar, 100 μm.

In glioma tissue, the level of Per2 mRNA was higher in the irradiated (10 Gy) group than in the control (untreated) group at 24 hours after irradiation (*p* < 0.05). The level of Per2 mRNA was lower in the Per2-knockdown group than in the control group at after 24 hours (*p* < 0.05) with or without irradiation (Figure 4A). A similar result was observed with protein level (*p* < 0.05) (Figure 4B). Comparing the unirradiated Per2 shRNA group with the unirradiated control group at the 24 hour time point the knockdown efficiency of Per2 was 54.56%. Furthermore, we measured the tumor volume of each irradiated group at the 24, 48 and 72 hour time points (Figure 3). Interestingly, tumor volumes were indistinguishable at 24 hours but expression levels of Per2 were different in each irradiated group. Although the expression of Per2 changes the growth of glioma, the tumor volume of each group may not differ because of the limited time and limited sensitivity of the gliomas at 24 hours (statistical difference was found in tumor volume at 48 and 72 hours). On the other hand, we found nuclear atypia and tumor-like morphology (Figure 5). Our tumor cells showed a large nucleus, hyperchromatism, and pleomorphism.

### Positive correlation between apoptosis and Per2 levels in glioma tissue

**Figure 6:**
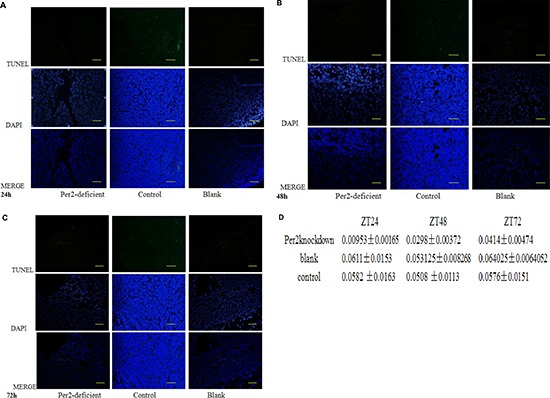
Apoptosis in cancer cells as assessed by TUNEL assay at (A) ZT24, (B) 48, and (C)72 Apoptotic cells were labeled with 3,3-diaminobenzidine using terminal deoxynucleotidyltransferase and counterstained with DAPI. Green fluorescence indicates positive staining for DNA strand breakage. Scale bar, 100 μm. *p < 0.05 **(D)** Levels of apoptosis as measured by TUNEL assay at each timepoint.

After 10 Gy of irradiation, the mice were sacrificed, and the internal organs were removed for further analysis. First, we measured apoptosis using a TUNEL (Terminal deoxynucleotidyl transferase dUTP nick end labeling) assay. The Per2-knockdown group exhibited an obvious trend of increased apoptosis over time (*P* < 0.05), while the other two groups showed little change in the levels of apoptosis (*P* > 0.05) (Figure 6).

### Positive correlation between DNA damage and Per2 levels in glioma tissue

**Figure 7:**
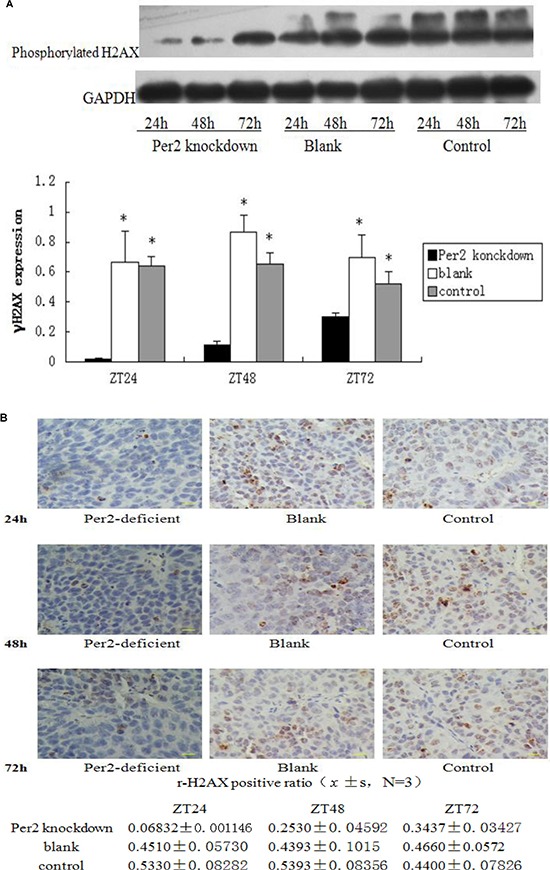
Tissue lysates from irradiated samples were analyzed by Western blot with antibodies against phosphorylated histone H2AX; cleaved GAPDH was used as an internal control (B) Immunohistochemistry staining of tumor samples. Blue speckles indicate normal cell nucleus and brown indicates positive cell nuclei which contain phosphorylated histone H2AX. 6 randomly selected 400× fields were counted, and mean H2AX + cells per field was obtained for statistical analysis. Scale bar, 100 μm. *p < 0.05.

X-ray exposure leads to breakage of double-strand DNA. We used western blotting for phosphorylated histone H2AX to identify DNA double-strand breaks. The Per2 knockdown group showed increasing DNA breakage over time while the blank and control virus groups were almost unchanged (Figure 7A). The histological result is consistent with the western blot result (Figure 7B). Blue speckles indicate normal cell nuclei and brown ones indicate positive cell nuclei with phosphorylated histone H2AX. 6 400 × magnification fields were randomly selected and counted; mean H2AX + cells per field was obtained for statistical analysis. Brown speckles in the Per2-knockdown group increased over time, while the other two groups were approximately equal (Figure 7B).

### Correlation between MDM2, c-myc, p53, ATM and Per2 expression levels in glioma tissue

**Figure 8:**
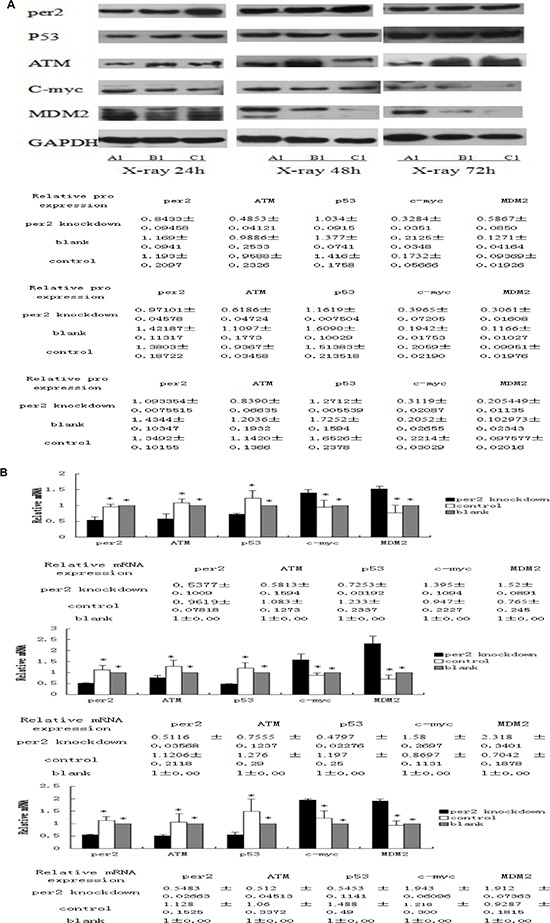
**(A)** Tissue lysates from irradiated samples were analyzed by Western blot with antibodies against per2 and downstream targets p53, ATM, Mdm2, and c-myc. Cleaved GAPDH was used as an internal control. *p < 0.05 Table indicates relative amounts of each protein as determined from densitometry. **(B)** Summary of three analyses on expression of c-myc, p53, ATM, Mdm-2, and Per2 mRNAs at ZT24,48, and 72 after X-ray irradiation. Each mRNA was quantified using qRT-PCR and cleaved GAPDH was used as an internal control. *p < 0.05

We examined the expression of ATM, TP53, MDM2, and C-MYC, important genes for repair, programmed cell death, and proliferation. Protein and phosphorylation levels were normalized to the level of GAPDH and baseline expression. After exposure to 10 Gy X-ray-irradiation, the expression of ATM and TP53 was reduced in Per2 knockdown U343 glioma cells relative to the other two groups at all measured time points (both *p* < 0.05). In contrast, the expression of the oncogenes c-myc and MDM2 increased in the irradiated shRNA-PER2 U343 glioma cells (Figure 8A). Differences in mRNA expression were found to correlate with similar changes in immunoreactive proteins detected by western blot (Figure 8B). *In vitro*, the same results were observed; downregulation of Per2 reduced the expression of ATM and TP53 and increased the expression of c-myc in X-ray-irradiated U343 glioma cells. Secondly, in the Per2 knockdown group, ATM and p53 proteins increased while Per2 and MDM2 were reduced over time. However, c-myc protein and mRNA were unchanged among the three time points.

## DISCUSSION

Mutations in the Per2 gene, an essential regulator of the mammalian circadian clock system, have been identified in a wide range of human cancers, including colorectal and breast cancer [26]. Furthermore, circadian Per2 disruption has been implicated in cell cycle dysfunction and apoptosis, which was evident by the aberrant rhythmic expression of the cell cycle gene cyclin D1 as well as the negative p53 regulator MDM2 [27]. Additionally, there are several links between Per2 and DNA damage responses. Aberrant Per2 expression results in potent downstream effects on both cell cycle and apoptotic targets, which is suggestive of a tumor suppressive role for Per2 [28]. Additional lines of evidence, suggest a role for Per2 in tumor suppression. Per2-deficient mice have a low tumor incidence; however, following γ-irradiation, these mice become cancer-prone [5]. In humans, Per2 expression is significantly reduced in both sporadic and familial primary breast cancers [7], and a few breast cancer cases contain PER2 mutations [29]. In cases where Per2 is not mutated, altered Per2 promoter methylation has been observed [6]. Consistent with this finding, Per2 expression is reduced in breast cancer stem cells (BCSCs) [30]. Several studies have described a correlation between Per2 and cell cycle regulation or DNA damage response gene expression [5, 11, 18, 31, 32].

In our previous study, we reported that Per1 and Per2 expression abnormalities are associated with glioma occurrence [9]. Another study showed that Per2 expression may increase the efficacy of radiotherapy against glioma [33]. Furthermore, the circadian genes Per1 and Per2 increase the radiosensitivity of gliomas *in vivo* [20]. In this study, we focused on how Per2 induces DNA damage and apoptosis of glioma cells after X-ray irradiation. Per2 knockdown in U343 glioma cells promoted the tumor formation process in nude mice, which is consistent with gastric cancer and breast cancer research [33, 34]. By irradiating glioma tissue with 10 Gy X-rays, we found that DNA damage and apoptosis of glioma cells were reduced in the Per2-knockdown group compared with the other groups. This result suggests that Per2 might be relevant in X-ray treatment, promote apoptosis of glioma cells, and be a suppressive gene.

Ionizing radiation is a genotoxic agent that produces a wide range of DNA alterations (e.g., strand breaks, base damage, and cross-links), which, after processing through the cellular repair machinery, determine the variety and severity of cellular and tissue effects. Double-strand breaks (DSB) are critical lesions that can lead to cell death or genomic instability unless properly repaired [35]. An important steps in evaluating the damage severity and cellular ability to advance through the cell cycle is the activation of p21WAF1/Cip1. p21 inhibits cell cycle-dependent kinases (CDK) by suppressing Cyclin E and Cyclin A-associated CDK2 activities, thus blocking cell cycle progression [36]. p21 acts as a cell cycle checkpoint and is able to block the cell cycle at both G1/S and G2/M phases. It is also one of the main factors that induce p53-dependent apoptosis [37, 38]. p21 is upregulated in the initial phases of human primary keratinocyte terminal differentiation and decreased at the late stages of the process [37]. This protein increase has been suggested as a necessary step in the removal of cells with accumulated DNA damage via apoptosis. When sub-lethal DNA damage is induced, p21 acts as an inducer of cell cycle arrest and facilitates damage repair [36–38]. Previous studies have used γH2AX foci as a sensitive monitor of DSB formation and repair in cultured cells and *in vivo* [39, 40]. We obtained tumor tissues from mice after a low dose of X-ray irradiation and detected DNA damage in glioma cells. Once a DNA strand break occurs, H2A is rapidly phosphorylated. In our study, we found that the level of phosphorylated histone H2AX in the gliomas of the Per2-knockdown group after X-ray irradiation was significantly lower than the control virus or normal control groups at all time points, indicating reduced DNA double strand breakage.

After X-ray irradiation, the expression of phosphorylated histone H2AX was increased in the Per2-knockdown group but unchanged in the control groups. Moreover, when we measured apoptosis in the three glioma groups after irradiation, we found that the apoptosis levels in the Per2-knockdown group were lower, consistent with the changes in DNA damage.

Previous studies have shown that Per-mutant mice are cancer-prone, whereas Cry1−/−; Cry2−/− mice are deficient in cell proliferation in the first 72 h of liver regeneration [5, 14, 41, 42,]. A similar deficiency in liver regeneration was reported for mice lacking the nuclear receptor FXR, which are also prone to spontaneous hepatocellular carcinoma [43, 44]. These findings suggest that cell proliferation is differentially controlled under different physiological conditions *in vivo*. Using the central clock-SNS-peripheral clock axis as a model system, we propose that central clock-controlled SNS signaling generates coupled AP1, peripheral clock, and ATM activation. AP1 activation leads to myc-induced cell cycle progression, while the activation of the peripheral clock inhibits myc overexpression and is required for ATM activity. ATM then induces p53 to prevent Myc oncogenic signaling by blocking p53-MDM2 interactions. Disruption of circadian rhythm desynchronizes the central clock-SNS-peripheral clock axis, which suppresses peripheral clock and peripheral clock-dependent ATM-p53 signaling; however, it has no effect on c-myc activation. Together, these events lead to Myc oncogenic activation, which promotes genomic instability and tumor development. This model suggests that the circadian clock plays a dual role in cell cycle control and suppression of tumor development by controlling homeostasis but not the inhibition of cell proliferation. Our finding regarding the sympathetic control of ATM-p53 signaling indicates that the induction of p53 occurs as an integrated part of the mammalian daily physiology *in vivo*. This finding has important therapeutic implications [45].

p53 is a potent transcription factor, activated in response to diverse stresses and environmental insults, leading to induction of cell cycle arrest, apoptosis, or senescence. Thus, the main function of p53 is to restrain the emergence of transformed cells with genetic instabilities, acting as the ‘guardian of the genome’. In normal cells, p53 is kept at low levels by MDM2, a ubiquitin ligase. MDM2 and p53 form a negative-feedback loop, in which p53 induces the expression of MDM2, which in turn promotes the degradation of p53 and quenches cellular p53 activity [46]. Approximately 50% of human cancers possess a mutated form of p53, while more than 17% of tumors exhibit mdm2 gene amplification. These alterations, separately or concomitantly, lead to poor prognosis and treatment failure [47–49]. For these reasons, the MDM2-p53 interaction appears to be an excellent target for cancer therapy and has been a focal point of research in both academia and industry, aiming to develop better targeted cancer therapeutics.

Per2 overexpression can lead to cell differentiation and apoptosis and changes the expression levels of apoptosis-related genes [18, 50]. In response to DNA damage, several checkpoint genes, including ATM/ATR and CHK1/2, are successively activated. Subsequent TP53 activation [51–55], results in cell cycle arrest at the G1/S, S, or G2/M phases, providing sufficient time for DNA damage repair [50, 51]. If DNA damage is not repaired in a timely manner, chromosomal deletions, duplications, or translocations can appear. MDM2 expression is induced by TP53, promoting p53 degradation and limiting its activity [46]. In our study, the Per2, P53, and ATM expression levels in the Per2-knockdown group were lower than those in the control virus and blank groups at 24, 48, and 72 hours after X-ray irradiation. However, MDM2 and c-myc expression was higher in the Per2-knockdown group. We believe that Per2 regulates glioma cell apoptosis via the ATM-p53 pathway, promotes ATM and P53 expression, and inhibits the c-myc and MDM2 expression. Per2 is an excellent potential target for glioma treatment.

We conclude that Per2 is both a tumor suppressor gene and an upstream regulatory gene of TP53. It inhibits tumor growth and promotes cancer cell apoptosis by regulating TP53 expression and DNA damage repair. Our findings identify Per2 as an excellent target for the clinical treatment of glioma and a reliable basis for post-radiation therapy and gene therapy for gliomas.

## MATERIALS AND METHODS

### Cell lines and reagents

The U343 glioma cell line was purchased from Ji Ni Biotechnology Co. Ltd. (Guangzhou, China). The cells were maintained according to the supplier's directions and were cultivated at 37°C in a 100% Rh 5% CO2/95% air atmosphere. ShRNA-PER2 lentivirus was obtained from the Genechem Chemical Technology Co. Ltd. (Shanghai, China). The oligonucleotide primer and probe sequences were designed and provided by Sangon Biotech Co. Ltd. (Shanghai, China). BALB/C mice were purchased from the Ningxia Medical University animal center. The per2, p53, c-myc, ATM, and mdm2 antibodies (Abcam, USA), the phosphorylated H2AX antibody (CST, USA), the total RNA extraction kit, the reverse transcription kit, the real time quantitative PCR kit, the total protein extraction kit, the protein concentration detection kit (Thermo-Fisher, USA), and the TUNEL kit (Roth, USA) were used according to the manufacturers’ instructions. A USA Varian IX (SNSS04) accelerator provided the 10 Gy X-ray irradiation.

### Transfection with shRNA lentivirus

Per2 expression levels were modified using a lentiviral transfection of shRNA (Table 1). U343 glioma cells were stably transformed with shRNA-PER2 and shRNA-control constructs according to the manufacturers’ protocol. U343 cells were plated in 6-well plates at a density of 2 × 104 cells/well in RPMI-1640 medium (Gibco, USA) and allowed to attach for 18 h prior to transfection with the shRNA lentivirus vectors. The viral constructs were permitted to integrate for 24 h after infection. Puromycin (3 μg/ml) was added to select cells with integrated retrovirus, and stable cell lines were established after 3 weeks. Then, qRT-PCR and Western blot analyses were used to verify the Per2 expression levels.

**Table 1:** Interference sequence of shRNA-PER2 are showed in table 1

Name	Plasmid sequences
Period2-RNAi	5ʹ–CCGGCCACGAGAATGAAATCCGCTACTCGAGTAGCGGATTTCATTCTCGTGGTTTTTG–3ʹ
Control-RNAi	5ʹ–TTTCTCCGAACGTGTCACGTTTCAAGAGAACGTGACACGTTCGGAGAATTTTTTC–3ʹ

### Animal experiments

These studies were conducted in accordance with the animal care guidelines of the Animal Studies Committee of Ningxia Medical University.

The three groups of cells were cultured and collected in the logarithmic growth phase. An appropriate volume of fetal bovine serum-free RPMI1640 medium and double antibody (Inactivated 10% fetal bovine serum and 1% mildew chain mildew) was added to each of the three groups of cells to bring them to a uniform concentration of 2 × 107 cells/ml. Samples were frozen for later establishment of transplanted tumor models. 54 BALB/C nude mice (male, 18–20 g) were divided into three groups that were administered one of the three different groups of cell. The mice were injected according to the SPF protocol, and tumor growth was observed. When the tumor volumes reached 1,000 mm3, each group was divided into irradiated and non-irradiated groups. The irradiated groups were administered a single 10-Gy X-ray dose. Three mice were sacrificed by cervical dislocation at 24, 48, and 72 hour time points; then, the tumor was excised.

### Tissue treatment

The tumors were harvested, and portions of the tumors were fixed in 4% formalin for 48 h. Morphological changes were evaluated by hematoxylin and eosin staining, while the remaining sample was cut into pieces for protein and RNA isolation.

### TUNEL analysis

Dewaxing and rehydration of the tissue sections was conducted according to standard protocols (i.e., heating at 60°C followed by washing in xylene and rehydration through a graded series of ethanol and double distilled water). The tissue sections were then incubated for 15–30 min at +21 to +37°C with a proteinase K working solution. The slides were then rinsed twice with PBS, and the area around the sample was dried. Then, 50 μl of TUNEL reaction mixture was added (a total volume of 50 μl of Enzyme solution (vial 1) was added to the remaining 450 μl of Label Solution in vial 2) to the sample. Samples were capped and incubated for 60 min at 37°C in a humidified atmosphere in the dark. The slides were rinsed 3 times with PBS. The samples were analyzed in a drop of PBS under a fluorescence microscope at this state, with 450–500 nm excitation and 515–565 nm emission detection (green).

### Histology analysis

Briefly, sections were deparaffinized, pretreated with 0.3% H2O2 for 20 min to inhibit endogenous peroxidase activity, blocked with 2% goat serum for 30 min, and incubated with a primary antibody overnight at 4°C. The avidin-biotin-complex technique and high-sensitivity diaminobenzidine (DAB+) chromogenic substrate system (Dako Denmark) were used for visualization; then, samples were counterstained with hematoxylin. We counted 6 randomly selected 400 × fields, and the mean number of H2AX+ cells per field was obtained for statistical analysis.

### Western blot analysis

Tumor extracts or cell lysates were mixed with sample loading buffer and separated under reducing conditions with a 10% SDS-polyacrylamide gel, then incubated with rabbit anti-per2, anti-mdm2, anti-p53, anti-ATM (Abcam), or anti-phosphorylated-H2AX (Ser139) antibodies (Cell Signaling Technology). Protein and phosphorylation levels were normalized to that of GAPDH (ProteinTech Group) and baseline expression.

### qRT-PCR analysis

The relative mRNA quantification of Per2 target genes was performed by RT-PCR as described above. Specific primers for p53, MDM2, c-myc, and ATM mRNA were designed to include intron/exon boundaries and are reported in Table 2. The relative expression of the Per2, p53, MDM2, c-myc, and ATM mRNA was determined using the relative quantification method and 2 -ΔΔCt analysis.

**Table 2:** Real-time RT-PCR: primer nucleotide sequences

Genes	Forward	Reverse
Per2	5ʹ–CCCTGGTGTCTGGGAAGAT–3ʹ	5ʹ–GGAGGTGAAACTGTGGAACA–3
ATM	5ʹ–TGTGACTTTTCAGGGGATTTG–3ʹ	5ʹ–ATAGGAATCAGGGCTTTTGGA–3ʹ
p53	5ʹ–CTCCTCAGCATCTTATCCGAGT–3ʹ	5ʹ–GCTGTTCCGTCCCAGTAGATTA–3ʹ
c-myc	5ʹ–CCTCCACTCGGAAGGACTATC–3ʹ:	5ʹ–TGTTCGCCTGACATTCTC–3ʹ
MDM2	5ʹ-GCTTTATGGGTGGATGCTGA–3ʹ	5ʹ-TTGCCTTTCGTTTGTTAGCTC–3ʹ
GAPDH	5ʹ-AGAAGGCTGGGGCTCATTTG-3ʹ	5ʹ-AGGGGCCATCCACAGTCTTC–3ʹ

### Statistical analyses

All data are presented as the mean ± SEM. Statistical analysis was performed with one-way analysis of variance (ANOVA) tests with Bonferroni's corrected *t*-tests for post-hoc pair-wise comparisons. Densitometric analysis of the immunoreactive bands was performed using the ImageJ program. For the *in vivo* experimental data, a two-way ANOVA was performed to compare the different parameters amongst the different groups. *P* < 0.05 was considered significant.
